# Supplementary figures and images for: Genome-wide identification and expression analysis of the MADS gene family in sweet orange (Citrus sinensis) infested with pathogenic bacteria
Source: PeerJ. 2024 Feb 29;12:e17001. doi: 10.7717/peerj.17001 (PMC10909352; doi:10.7717/peerj.17001)

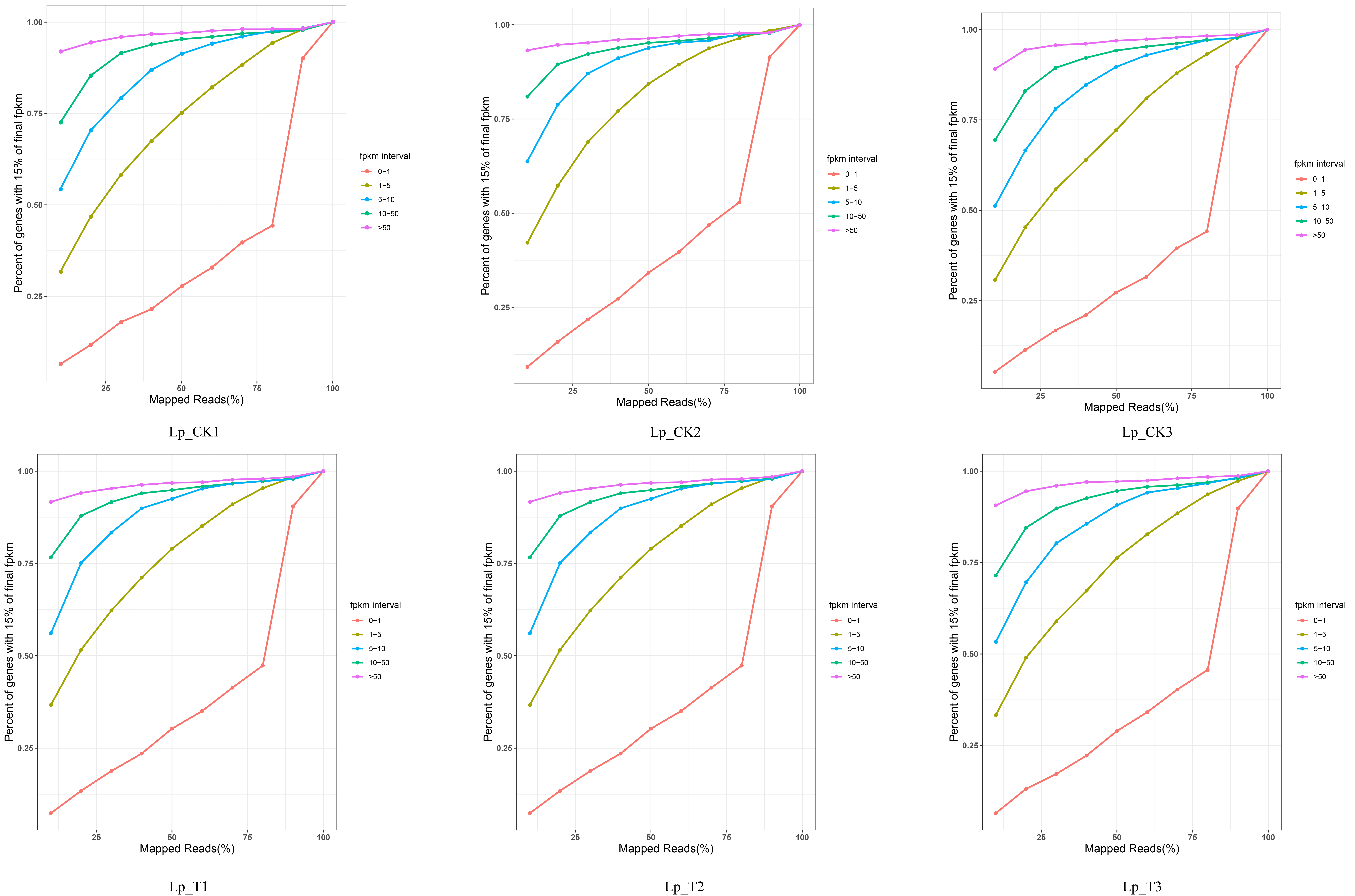

Supplement: Figure S1 [file peerj-12-17001-s001.png]

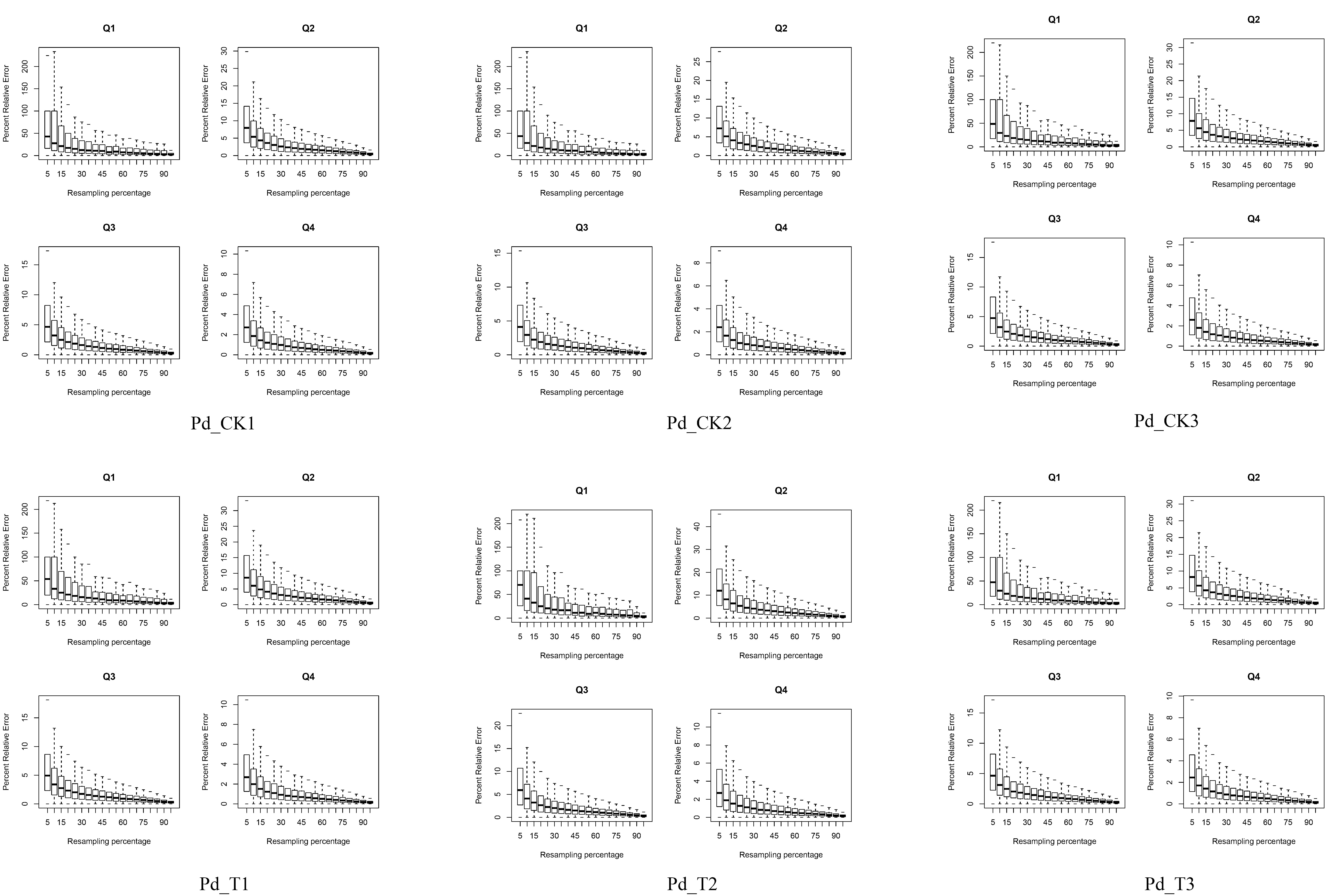

Supplement: Figure S2 [file peerj-12-17001-s002.png]
